# Molecular characterization of isolated infectious bronchitis viruses from affected vaccinated broiler flocks in Syria

**DOI:** 10.1186/s12917-020-02672-1

**Published:** 2020-11-19

**Authors:** Tamara Al-Jallad, Morshed Kassouha, Mohamad Salhab, Anouar Alomar, Mouhamad AL-Masalma, Fahim Abdelaziz

**Affiliations:** 1 The General Commission for Scientific Agricultural Research, Lattakia, Syria; 2 Department of Animal Production, Tishreen University, Lattakia, Syria; 3 Department of Microbiology, Faculty of Veterinary Medicine, Hama University, Hama, Syria; 4 Faculty of Sciences, Al-Baath University, Homs, Syria; 5 Department of Molecular Biology, Ministry of Agriculture, Damascus, Syria; 6 Faculty of Sciences, Tartous University, Tartous, Syria

**Keywords:** Infectious bronchitis virus, Molecular characterization, Broiler, Syria

## Abstract

**Background:**

Avian Infectious Bronchitis Virus (IBV) is a highly contagious disease that imposes a huge economic burden on the global poultry industry. IBV contains numerous serotypes and variants with incomplete tenuous cross immunological protection. The failure of currently used vaccines to protect against diverse, circulating IBV strains that are specific to a given region poses a major problem for the poultry industry. Thus, there is an urgent need to conduct studies aimed at genotyping field IB viruses. In this study, we have determined the molecular characteristics of circulating IBV by sequencing the S1 gene of viral isolates from affected previously vaccinated broiler flocks suffering from the disease.

**Results:**

Ten isolates propagated in embryonated eggs showed an ability to induce typical IBV lesions after three successive viral passages. We performed a nested RT–PCR assay that targeted the hypervariable region 3 (HVR-3) of the S1 gene, and identified the isolates as IBV through sequence analysis. The IBV isolates showed sequence similarity between the Syrian isolates that vary from 96.20 to 100%, and those being closer to the Variant-2 strain IS/1494/06 (EU780077.2) with 97.5 to 99.4% similarities. However, less nucleotide identity was found with sequences belonging to the used vaccine strains such as H120, Mass5, and 4/91.

**Conclusions:**

This study showed the presence of the Variant-2 strain circulating in Syrian broiler flocks showing signs of IBV disease. Currently, there is no commercial effective vaccine which protects birds against the Variant-2 strain. Continuous monitoring procedures should be taken to control and limit the spread of the IBV Variant-2 strain. This research emphasizes both the importance of epidemiological monitoring in intensive poultry farming for novel pathogens and the use of local isolates as future vaccine targets.

## Background

Avian infectious bronchitis (IB) is an acute and highly contagious disease caused by the avian infectious bronchitis virus (IBV) [1, 2]. IBV is an enveloped and pleomorphic RNA virus (27.6 kb single-stranded positive sense genome) in the genus *Gammacoronavirus* of the *Coronaviridae* family [3, 4]. The viral genome encodes an RNA-dependent RNA polymerase (RdRp), accessory and regulatory proteins, and four structural proteins: the spike proteins (S), the membrane protein (M), the internal nucleoprotein (N), and the envelope protein (E) [5]. IBV was first reported in North Dakota, USA, by Schalk and Hawn 1931 [6] as a novel respiratory disease affecting chickens. The virus is acquired following inhalation or direct contact with contaminated poultry, litter, animal husbandry equipment, or other fomites [7]. It mainly affects the respiratory tract, the kidney, and the reproductive system of chickens across all age groups [2]. Birds infected with IBV show clinical signs like sneezing, tracheal coarse crackles, coughing, and reduction in feed intake [8]. IB morbidity and mortality cause heavy economic losses throughout the poultry industry around the world [9], with impacts that extend vertically to the eggs of chickens affected by IBV. Chickens infected with IBV have decreased egg production and quality as a result of thin, fragile, misshapen shells, and thin watery eggs [5, 10].

The disease is conventionally diagnosed by different methods, including viral isolation (VI), followed by viral neutralization test (VNT), agar – gel precipitation test (AGPT), reverse transcriptase- polymerase chain reaction (RT-PCR) [11], restriction fragment length polymorphism (RFLP) [12], and real-time PCR [13]. Common indirect methods include the use of Enzyme-Linked Immuno-Sorbant Assay (ELISA) for the detection of specific antibodies.

RT-PCR is the most widely used molecular technique to detect IB viral genome directly from tissue samples or from allantoic fluid from Specific-Pathogen-Free embryonated chicken eggs (SPFEE) inoculated with field suspected samples [14].

It became known to all that PCR-based techniques are both fast and sensitive in comparison with classical detection methods [15]. Spike glycoprotein (S), the major protein of IBV, is anchored in the viral envelope and cleaved into two proteins, S1 and S2, during the post-translational phase [16, 17]. The S1 protein has three hypervariable regions (HVRs) that are targets for neutralizing antibodies and are the main determinants for serotype specificity [16]. Variation in these epitopes is a cause of weakened vaccine-induced immunity [16, 18], as sequence variations in S1 protein, used in the determination of new viral genotypes, are also possibly involved in antiviral response [18]. Such protein is a differentiating factor between IBV strains and thus is a main target for genotype characterization, and also plays an important role in attachment and viral entry into cells through the sialic acid receptor [19]. Amino acid variation in the glycoprotein S1 has an important role in tissue tropism and IBV virulence [20], and the RT-PCR amplification and sequencing of all or part of the S1 gene, allows identification of IBV strains [17]. Special site changes in the amino acid sequence of the S protein could lead to antigenic variation and a new type of the virus, which may be different from the vaccine strains currently used in immunization programs [18, 21], and may require further development of a homologous vaccine.

Recently, Valastro et al. [18] defined IBV strains into six genotypes comprising 32 distinct viral lineages that include several unassigned recombinants of inter-lineage origin based on the complete S1 gene sequence. The distribution and diversity of these IBV lineages differ with geographic location [18]. Some lineages are ubiquitous, and the global distribution of major IBV serotypes has been shown by Bande et al. (2017) [22] such as Mass-type, 4/91 (793B or CR88)-like, D274-like (D207, D212 or D1466, D3896), D3128, QX-like, and Italy02 [22]. In contrast, some lineages are spread in specific geographical areas, such as GI-23 lineage, which is confined to the Middle East and includes Variant-2 viruses [18].

The main problem in the control of infectious bronchitis is the ability of the virus to generate antigenic variants, due to mutation, insertion, deletion, and/or recombination of the S1 spike genes from two different viruses during mixed infection [16].

In 2018 and 2019, we undertook a nested RT-PCR followed by sequencing to detect IBV in ten commercial poultry flocks in Syria that presented clinical signs possibly associated with IBV. The main aim of this study was to detect the major IBV genotype variants circulating in poultry flocks in Hama, Homs, and Tartaus Governorates, and to monitor the possible emergence of any new IBV genotypes. This could provide a guide to the optimal use of existing vaccines, and to the development of new vaccines or vaccine strategies.

## Results

### Virus isolation

**Fig. 1:**
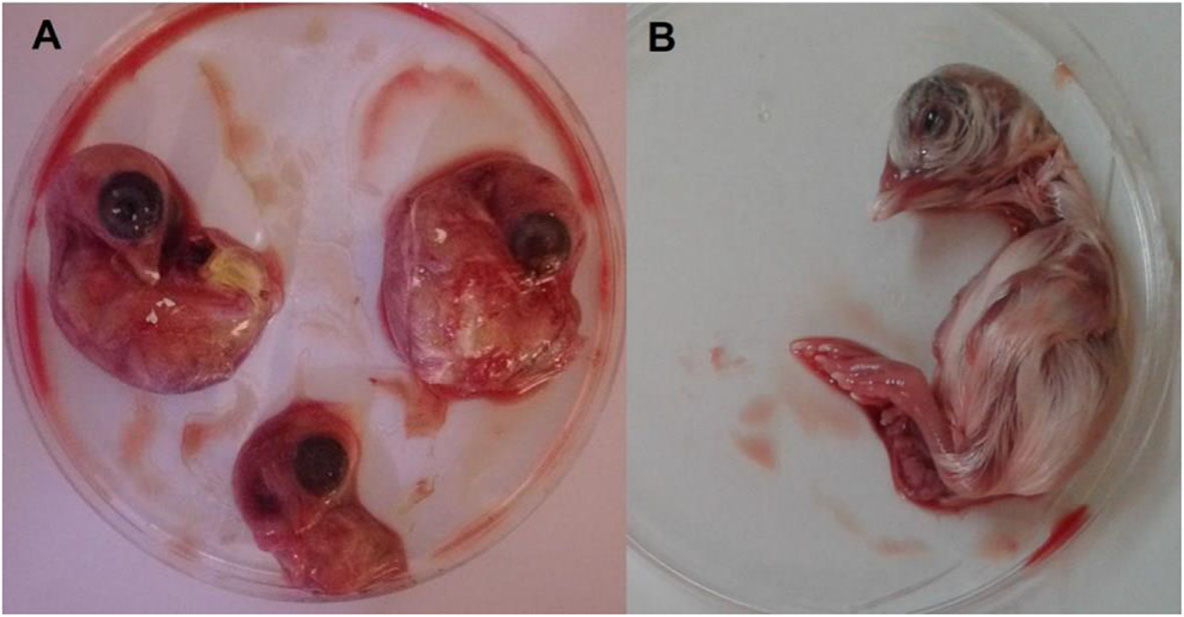
**a**Stunting embryos (16 days). **b** Control (16 days)

SPF embryonic eggs were passaged with filtrate of trachea or kidney from chickens exhibiting symptoms of IB. After the third viral passage, typical IBV hemorrhagic embryonic lesions and delay of development and growth were observed in all eggs. The embryos exhibited a wrinkled and folded appearance, and atypical wrapping of fingers (Fig. 1). In some cases, the inoculation resulted in embryonic death.

### Nested RT-PCR

**Fig. 2:**
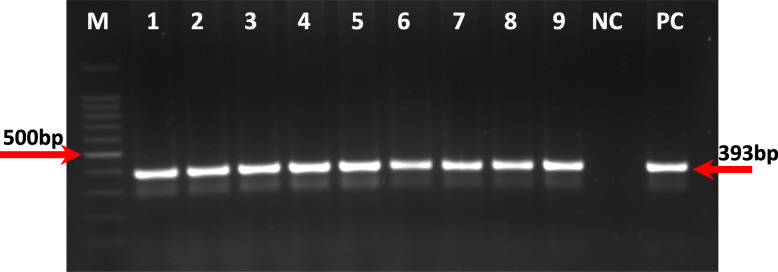
Agarose gel electrophoresis of Nested PCR products ≈ 393 bp. Lane M: 100 bp ladder, Lanes 1–9 Field Isolates (Lane1:T1T, Lane2:Ho1T,Lane3:T2k, Lane4:T3T, Lane5:T4T, Lane6:T5T, Lane7:Ha2T, Lane8:T6K, Lane9:T7K, and Isolate Ha1T not included ). NC Negative control (nuclease-free water). PC Positive control (H120 vaccine strain)

Ten tissue samples were collected from the trachea (*N* = 7) or kidney (*N* = 3) of chickens suspected to have IBV. We performed nested RT-PCR on all ten field samples to amplify a 393 bp amplicon from the partial spike glycoprotein of the S1 subunit fragment gene. All ten field samples produced the expected 393 bp amplicon after nested RT-PCR (Fig. 2).

### Sequencing of partial S1 gene PCR products

All fragments obtained after nested RT-PCR were sequenced and the isolates were aligned with sequences from Genbank using BLAST (WWW. ncbi. nlm. nih. gov/ Blast). BLAST alignment indicated that our sequences shared 97.5–99.4% identity with the Variant-2 IS/1494/06 (EU780077.2). All sequences were submitted to GenBank of NCBI database (Accession numbers MT010126, MT010127, MT010128, MT010129, MT010130, MT010131, MT010132, MT010133, MT010134 and MT010135) (Table 1).

**Table 1:** Nucleotide identities (%) and amino-acid similarities (% in brackets) of partial S1 gene of ten Syrian IBV isolates with reference IBV strains

		1	2	3	4	5	6	7	8	9	10	11	12	13	14	15	16	17
1	**Ha1T**^**b**^**(MT010135)**^**a**^	-	99.7 (99.0)	99.7 (99.0)	99.7 (95.2)	99.6 (100)	99.4 (98.1)	100.0 (100)	96.8 (93.3)	100.0 (100)	99.4 (100)	99.4 (98.1)	89.5 (87.5)	99.0 (97.1)	82.3 (83.7)	82.5 (82.7)	81.8 (79.8)	81.8 (79.8)
2	**T1T**^**b**^**(MT010132)**^**a**^		-	99.3 (98.0)	99.7 (99.0)	99.3 (98.9)	99.0 (97.0)	99.7 (99.0)	97.0 (93.3)	99.7 (99.0)	99.0 (99.0)	99.3 (98.0)	89.0 (86.9)	99.0 (97.0)	81.7 (81.8)	81.9 (80.8)	80.9 (77.8)	80.9 (77.8)
3	**Ho1T**^**b**^**(MT010133)**^**a**^			-	99.7 (99.0)	99.3 (98.8)	99.0 (97.0)	99.7 (99.0)	97.3 (94.9)	99.7 (99.0)	99.0 (99.0)	99.3 (98.0)	89.7 (87.9)	99.0 (97.0)	81.4 (82.8)	82.1 (81.8)	81.1 (78.8)	81.1 (78.8)
4	**T2K**^**b**^**(MT010131)**^**a**^				-	99.6 (100)	99.0 (93.3)	99.7 (95.2)	96.5 (89.4)	99.7 (95.2)	99.0 (95.2)	99.0 (94.2)	89.2 (83.7)	98.7 (93.3)	82.0 (78.8)	82.2 (77.9)	81.6 (75.0)	81.6 (75.0)
5	**T3T**^**b**^**(MT010130)**^**a**^					-	98.9 (97.8)	99.6 (100	96.4 (93.4)	99.6 (100)	99.3 (100)	98.6 (98.9)	89.9 (89.5)	98.3 (97.9)	83.5 (84.0)	83.3 (83.2)	81.5 (80.0)	81.4 (79.6)
6	**T4T**^**b**^**(MT010129)**^**a**^						-	99.4 (98.1)	96.2 (91.3)	99.4 (98.1)	98.7 (98.1)	98.7 ( 96.2)	88.9 (85.6)	98.4 (95.2)	82.3 (83.7)	82.5 (82.7)	81.2 (77.9)	81.2 (77.9)
7	**T5T**^**b**^**(MT010128)**^**a**^							-	96.8 (93.3)	100.0 (100)	99.4 (100)	99.4 (98.1)	89.5 (87.5)	99.0 (97.1)	82.3 (83.7)	82.5 (82.7)	81.8 (79.8)	81.8 (79.8)
8	Ha2Tb(MT010134)*								-	96.8 (93.3)	96.2 (93.3)	97.5 (95.2)	88.2 (85.6)	97.1 (94.2)	80.6 (80.8)	82.8 (82.7)	80.9 (77.9)	80.9 (77.9)
9	**T6K**^**b**^**(MT010127)**^**a**^									-	99.4 (100)	99.1 (98.1)	89.3 (87.7)	98.7 (97.2)	82.5 (84.0)	82.7 (83.0)	82.1 (80.2)	82.1 (80.2)
10	**T7K**^**b**^**(MT010126)**^**a**^										-	98.7 (98.1)	89.5 (87.5)	98.4 (79.8)	82.3 (83.7)	81.8 (82.7)	81.8 (79.8)	81.8 (79.8)
11	**IS/1494/06 ( EU780077)**^**a**^											-	88.3 (88.8)	99.5 (98.1)	82.3 (82.2)	79.1 (83.2)	79.9 (78.5)	81.4 (78.5)
12	**IS/885 (AY279533.1)**^**a**^												-	91.1 (88.3)	82.8 (80.6)	79.0 (81.1)	78.6 (76.6)	80.7 (76.6)
13	**Egypt/Beni-Suef/01(JX174183)**^**a**^													-	81.6 (81.7)	80.5 (82.5)	81.4 (79.1)	80.9 (78.5)
14	**MHW-QX-KDL-3-2012 (MH671340.1)**^**a**^														-	82.0 (84.3)	80.2 (73.6)	78.6 (74.8)
15	**strain 4/91 ( KF377577.1)**^**a**^															-	77.3 (74.6)	77.6 (73.8)
16	**H120 ( KF188436.1)**^**a**^																-	100 (100)
17	**Ma5 ( KU736747 )**^**a**^																	-

^a^GenBank accession numbers

^b^Sequenced in this study

### Phylogenetic analysis of partial S1 subunit

**Fig. 3:**
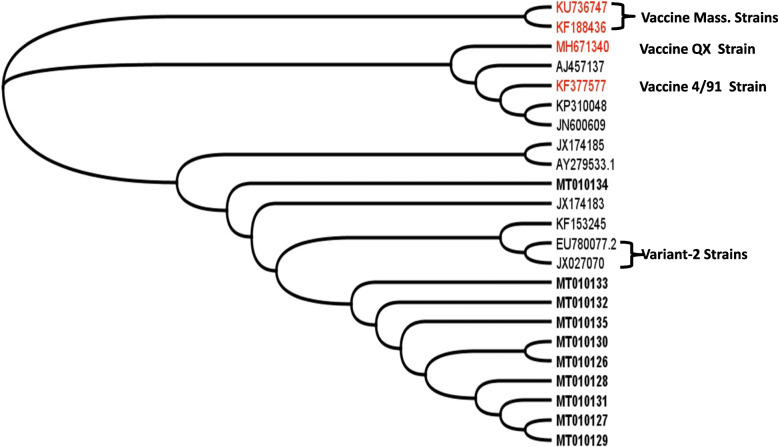
Phylogenetic tree based on alignment of partial S1 gene sequences of ten Syrian IBV variants (bold line), vaccine strains (red line) and seventeen Isolates/strains retrieved from GenBank. The phylogenetic tree was constructed using of Geneious 4.8.4 software with neighbor-joining method using of 500 bootstrap replicates

From our ten sequences, we constructed a phylogenetic tree using Geneious 4.8.4 software (Fig. 3) that revealed three distinct clusters: Cluster I included all ten Syrian isolates, Egyptian Variant 1 strain (JX174183), Egyptian Variant 2 strain, Israeli IS/1494/06 Variant 2 (EU780077.2) and IBV/chicken/Kurdistan Erbil/12VIR10065-16/2012 (KF153245). Cluster II included an Italy-02 strain, two Iranian strains (strain IR-Razi-HKM1-2010 and strain Iran/793B/H741/13), vaccine strains 4/91 and MHW-QX-KDL-3-2012. Finally, Cluster III consisted of the two Massachusetts vaccine strains (Ma5, H120).

The present study showed high sequence similarities (96.2–100%) among Syrian isolates, which appear to be closer to strains that belong to the Variant-2 (EU780077.2 and JX027070.). A lower percentage of nucleotide identity was found with the vaccine strains H120, Mass5, and 4/91.

## Discussion

IBV is a major pathogen in both vaccinated and non-vaccinated stock within the poultry sector. In recent years, IBV has caused significant economic losses in Syrian poultry production. IBV-based vaccination strategies targeting Ma5, H120, (and 4\91in some flocks) have been applied for IB control in Syrian poultry farms. However, IB-suspected cases have been frequently reported from broiler flocks, and were attributed to loss of protection after initial vaccine effectiveness or host’s inability to respond to primary vaccination.

There are two hypothetical reasons for primary vaccine failure: the first is the emergence of new variants, and the second is weak or no cross-protection between the field viruses and the strain used in vaccination. This study has proven the widespread of the IB disease among vaccinated broiler flocks and the circulation, in the Syrian regions of Hama, Homs, and Tartous, of genetically new isolates different from the used vaccine strains (H120 and Ma5).

We analyzed the partial S1 gene of all these IBV isolates and compared them with some sequence information available in GenBank. Phylogenetic analysis revealed that the Syrian IBVs isolates are closely related to Variant-2 strain. The Variant-2 strain is the most common strain in all countries which border Syria, and in the Middle East [23–25]. The Egypt/Beni-Suef/01 isolate was detected in Egypt with 99.5% similarity with IS/1494/06 [26]. In Libya, sequences of the S1 gene of IBV strains obtained from broiler flocks with respiratory symptoms of IB formed a cluster with 100% relatedness to the Variant-2 strain (IS/1494/06) [8]. Kahya et al. reported that eight Turkish IBVs isolates from broiler and breeder chicken flocks were related to the same Variant-2 IS/1494/06 IBV strain (EU780077), and showed 99% nucleotide similarity [23]. The Syrian isolates were related to the same Israeli Variant-2 strain with 97.5–99.4% similarity.

We found that the Syrian isolates shared between 96.2 and 100% nucleotide similarity with each other. and it is very interesting to be noted here that the sequence of the Ha2T sample was comparatively more genetically divergent from the rest of Syrian samples, sharing between 96.2 and 97.3% sequence similarity.

The virus isolated from the sample Ha2T needs to be further analyzed and a full S1 gene sequencing realized to get complete answer about these differences. Even though detection of mutations or recombination may exist, the isolate seems to be closer to Variant-2, when amino acid sequences were compared.

The samples studied here belong to GI-23 lineage that represents a unique wild-type cluster of IBV viruses, geographically confined to the Middle East [18]. Strains belonging to this lineage have been detected since 1998 in Middle East countries and are actively circulating [24]. Some have become dominant in the majority of farms and are involved in respiratory and renal lesions [27]. Previously, many of these strains were assigned Variant-2 to distinguish them from those clustering within Variant-1 [28, 29].

Currently, the major control measures of IB in broiler flocks are based on vaccination with live attenuated IBV vaccine (Mass serotypes) such as H120, and Ma5 strains. Chicken flocks vaccinated with Mass serotype live vaccines usually failed to present full protection against virulent wild-type IBVs, resulting in high IB-induced morbidity and mortality within vaccinated chicken flocks. The low correlation in nucleotide similarity between the ten IBV isolates in this study and the vaccine strains (average nucleotide identity of 81.5%) (Table 1) could explain the failure of the H120 and Ma5 vaccination programs to control IBV in these flocks. Awad et al., (2015) showed that vaccination with (H120) strain of one-day old chicks followed by a second, but different vaccination at day 14 (4/91) may provide 80% protection against Variant-2 strains [30]. However, based on the genetic differences between the Syrian isolates presented in this study and the vaccine strains, a more efficacious vaccine strategy may be used to generate an IBV vaccine that is homologous to the Variant-2 strains found within the Middle East.

Although, there is no commercially available vaccine against Variant-2 strains. Local trial vaccines were recently developed and evaluated in Egypt and Iran with promising results [31, 32].

## Conclusions

In this study, we conducted partial sequencing of the S1 gene of Syrian isolates sampled from chickens suspected to carry IBV. We contextualized the genetic sequences of the Syrian strains in a phylogenetic analysis with other worldwide reported isolates, including strains that are commonly used in IBV vaccines. The Syrian variants were relatively close to Variant-2 strains, which belong to GI-23 lineage. Further epidemiological surveillance studies are needed in order to explain the mechanism of genetic variation in these emerging pathogens and their biological properties, including their pathogenicity. There is a substantial need to develop a vaccine that protects broiler flocks against infection with Variant-2 IB viruses. The continuous characterization of new IBV strains is important for understanding the molecular evolution of different genotypes and for selecting candidate virus strains for vaccine strain development.

## Methods

### Tissue samples

Trachea and kidney samples were collected from suspected IBV outbreaks in Hama, Homs, and Tartaus governorates in Syria. All flocks were vaccinated as referred in (Table 2), and suffered from respiratory signs as gasping, sneezing, and rales. The most commonly observed necropsy lesions included serous, or caseous exudate in the trachea, and cloudiness in the air sacs. A caseous plug was found in the lower trachea or bronchi in all dead birds. Three flocks were suspected to be infected with nephropathogenic IBV strain, as the kidneys were swollen and whitish with the tubules often distended with urates. The samples were denoted by a short name starting with the first letters of the name of the governorate, followed by a number indicating its order, and ending with a letter indicating the tissue sampled. For example, sample Ha1T was from Hama Governorate, No.1, and was collected from trachea. Sample collection was performed upon farm owner’s permission.

**Table 2:** Details of IBV samples obtained in the current study, during 2018-2019

Sample	Governorate	^**a**^Age (day)	Vaccine	Organ of isolation
Ha1T	Hama	27	Ma5	Trachea
T1T	Tartous	37	H120	Trachea
Ho1T	Homs	33	H120	Trachea
T2K	Tartous	29	H120+Ma5	Kidney
T3T	Tartous	34	H120+Ma5	Trachea
T4T	Tartous	28	H120	Trachea
T5T	Tartous	27	H120	Trachea
Ha2T	Hama	35	H120	Trachea
T6K	Tartous	30	H120	Kidney
T7K	Tartous	35	H120	Kidney

^a^Age: bird's age at sample collection

### Virus isolation and propagation

All the samples were homogenized in laboratory mortar after adding two volumes of 0.9% NaCl normal saline to one volume of tissue. Subsequently, samples were centrifuged at 1500 g for 20 min at 4 °C. The supernatant was filtered through a 0.22um filter; the filtrate was supplemented with 1000 IU /ml of penicillin and 10 mg/ml of streptomycin, and 200 µl of this homogenate was inoculated into the allantoic cavity of 10-day-old fertile SPF embryonated eggs. Three eggs were used for each sample.

The inoculated eggs were incubated at 37 °C and candled daily to check for embryonic viability. After 5–6 days of incubation, the chorioallantoic fluid was harvested and used for subsequent passages. Three serial passages were performed, and the chorioallantoic fluid was collected 5–6 days post-inoculation. In addition, two uninoculated SPF eggs were used as controls in every isolation passage process [33].

### Viral RNA extraction and RT-PCR technique

The collected samples (trachea and kidney) were stored at − 20 °C until RNA extraction was performed. RNA extraction was carried out from tissue using GF-1 Total RNA extraction kit (Vivantis, Malaysia) according to the manufacturer’s instructions. RNA extraction was followed by RT-PCR.

Reverse transcription (RT) was performed using a two-step RT-PCR Kit (Vivantis, Malaysia). Briefly, RNA was added to a primer mixture containing.

7 µL total RNA, 1 µL gene-specific primer SX2-, and 1 µL 10 mM dNTPs mix. The reaction was incubated at 65 °C for 5 minutes, followed by chilling in ice for 2 minutes, and then cDNA synthesis mix was added (2 µL10x Buffer M-Mul V, 0.5 µL M-Mul V reverse transcriptase enzyme and nuclease-free water to a final volume of 25 µL). The reaction mix was then incubated at 42 °C for 60 min, and at 85 °C for 5 min to stop the reaction. 8 µL of cDNA was used as template for PCRs.

### Nested PCR

Primary PCR was performed according to the methods in [Worthington et al. 2008] [10]. The primary PCR was conducted using a pair of specific primers: SX1+ (5’-CACCTAGAGGTTTGT/CTA/TGCAT-3’) and SX2- (5’-TCCACCTCTATAAACACCC/TTT-3’) (from Metabion-Germany) in a final volume of 25µL containing: 8 µl of cDNA, 0.5µL of each primer (10 mM), 12.5µL of master mix (One PCR, Gene Direx), and 3.5 µL nuclease-free water. The mixture was placed in a thermal cycler (Techne-512) according to the following program: one cycle of initial denaturation at 94 °C for 2 min and 35 cycles of the following steps: denaturation at 94 °C for 30 sec, annealing at 56 °C for 30 sec, and extension at 72 °C for 1 min. Then one cycle of final extension was performed at 72 °C for 7 min. A nested PCR assay, using the specific primers SX3+ (5’-TAATACTGGC/TAATTTTTCAGA-3’), SX4- (5’AATACAGATTGCTTACAACCACC-3’) (from Metabion-Germany) was carried out to amplify a segment of ≈ 393-bp of the S1 gene. 1µL of the primary PCR product was used as a template in the nested PCR. The same methodology that was used in the primary PCR reaction was used in the nested PCR reaction, with the exception that annealing temperature was 53 °C. The H120 vaccine strain was used as a positive control for RNA extraction and amplification reactions, and nuclease-free water was used as a negative control. The nested PCR product products were visualized by electrophoresis on a 1.5% agarose gel stained with ethidium bromide.

DNA products were gel-purified using an GF-1 AmbiClean Kit (Vivantis, Malaysia), according to the manufacturer’s instructions, and the concentration of the purified DNA was then determined using a Nanodrop instrument (Thermo). The purified DNA was stored at -20 °C until sequencing.

### Sequencing and sequence analysis

The purified nested PCR products of the partial S1 gene were sequenced via a Macrogen Sequencing System (Macrogen Co., Korea).

Sequences were aligned with each other using Geneious v 4.8.4 sequence analysis software. The obtained sequences were subjected to BLAST analysis using the BLAST Tool at NCBI GeneBank (Basic Local Alignment Search Tools) (http://www.ncbi.nlm.nih.gov/blast). All ten sequences of this work were submitted to the NCBI GenBank database (accession numbers in Table 1). Nucleotide sequences of 13 reference strains were retrieved from the GeneBank database (Fig. 3) and integrated with our sequences to construct a phylogenetic tree .

### Phylogenetic analysis

The phylogenetic tree was constructed with Geneious 4.8.4 software using a neighbor-joining method and 500 bootstrap replicates. This phylogeny analysis included the sequences of ten Syrian IBV isolates, four vaccine strains, and nine foreign isolates from Middle East and Italy.

## Data Availability

The datasets generated and/or analysed during the current study are available in the National Center for Biotechnology Information (NCBI) repository, under these GenBank accession numbers MT010126, MT010127, MT010128, MT010129, MT010130, MT010131, MT010132, MT010133, MT010134 and MT010135.

## Abbreviations

IB: Infectious Bronchitis
IBV: Infectious Bronchitis Virus
PCR: Polymerase Chain Reaction
HVRs: Hypervariable Regions
RNA: Ribonucleic Acid
RT-PCR: Reverse Transcriptase Polymerase Chain Reaction
cDNA: Complementary DNA
S: Spike

## References

[1] Sjaak de Wit JJ, Cook JK, van der Heijden HM (2011). Infectious bronchitis virus variants: a review of the history, current situation and control measures. Avian Pathol.

[2] Shyma K, Sankar S, Aravindakshan T, Krithiga K, Bosewell A, Sarika N, Mini M (2018). Isolation and Molecular Detection of Infectious Bronchitis Virus Isolates from Chicken. Int J Curr Microbiol App Sci.

[3] King AM, Lefkowitz E, Adams MJ, Carstens EB: Virus taxonomy: ninth report of the International Committee on Taxonomy of Viruses, vol. 9: Elsevier; 2011.

[4] Xu C, Zhao J, Hu X, Zhang G (2007). Isolation and identification of four infectious bronchitis virus strains in China and analyses of their S1 glycoprotein gene. Vet Microbiol.

[5] Cavanagh D, Naqi SA, Saif YM, Barnes HJ, Glisson JR, Fadly AM, McDougald LR, Swayne DE (2003). nfectious bronchitis. Diseases of poultry.

[6] Schalk A (1931). An apparently new respiratory disease of baby chicks. J Am Vet Med Assoc.

[7] Jahantigh M, Salari S, Hedayati M (2013). Detection of infectious bronchitis virus serotypes by reverse transcription polymerase chain reaction in broiler chickens. SpringerPlus.

[8] Awad AM, Sediek ME, El-Yamany ME. Isolation and Molecular Characterization of Novel IBV Isolates from Broiler Chicken Farms in Egypt. Alexandria J Vet Sci 2014, 42(1).

[9] Feng K, Wang F, Xue Y, Zhou Q, Chen F, Bi Y, Xie Q (2017). Epidemiology and characterization of avian infectious bronchitis virus strains circulating in southern China during the period from 2013–2015. Sci Rep.

[10] Worthington KJ, Currie RJ, Jones RC (2008). A reverse transcriptase-polymerase chain reaction survey of infectious bronchitis virus genotypes in Western Europe from 2002 to 2006. Avian Pathol.

[11] De Wit JJ (2000). Detection of infectious bronchitis virus. Avian Pathol.

[12] Kwon HM, Jackwood MW, Gelb J (1993). Differentiation of infectious bronchitis virus serotypes using polymerase chain reaction and restriction fragment length polymorphism analysis. Avian Dis.

[13] Meir R, Maharat O, Farnushi Y, Simanov L (2010). Development of a real-time TaqMan® RT-PCR assay for the detection of infectious bronchitis virus in chickens, and comparison of RT-PCR and virus isolation. J Virol Methods.

[14] Najafi H, Ghalyanchi AL, Hashemzadeh M, Madadgar O, Karimi V, Farahani R, Abdollahi H, Maghsoudsloo H, Seifouri P (2016). Pathogenicity characteristics of an Iranian variant-2 (IS-1494) like infectious bronchitis virus in experimentally infected SPF chickens. Acta Virol.

[15] Liu HJ, Lee LH, Shih WL, Lin MY, Liao MH (2003). Detection of infectious bronchitis virus by multiplex polymerase chain reaction and sequence analysis. J Virol Methods.

[16] Naguib MM, Höper D, Arafa A-S, Setta AM, Abed M, Monne I, Beer M, Harder TC (2016). Full genome sequence analysis of a newly emerged QX-like infectious bronchitis virus from Sudan reveals distinct spots of recombination. Infect Genet Evol.

[17] Jackwood MW, Hall D, Handel A (2012). Molecular evolution and emergence of avian gammacoronaviruses. Infect Genet Evol.

[18] Valastro V, Holmes EC, Britton P, Fusaro A, Jackwood MW, Cattoli G, Monne I (2016). S1 gene-based phylogeny of infectious bronchitis virus: an attempt to harmonize virus classification. Infect Genet Evol.

[19] Belouzard S, Millet JK, Licitra BN, Whittaker GR (2012). Mechanisms of coronavirus cell entry mediated by the viral spike protein. Viruses.

[20] Madu IG, Chu VC, Lee H, Regan AD, Bauman BE, Whittaker GR (2007). Heparan sulfate is a selective attachment factor for the avian coronavirus infectious bronchitis virus Beaudette. Avian Dis.

[21] Adzhar A, Gough R, Haydon D, Shaw K, Britton P, Cavanagh D (1997). Molecular analysis of the 793/B serotype of infectious bronchitis virus in Great Britain. Avian Pathol.

[22] Bande F, Arshad SS, Omar AR, Hair-Bejo M, Mahmuda A, Nair V (2017). Global distributions and strain diversity of avian infectious bronchitis virus: a review. Anim Health Res Rev.

[23] Kahya S, Coven F, Temelli S, Eyigor A, Carli KT (2013). Presence of IS/1494/06 genotype-related infectious bronchitis virus in breeder and broiler flocks in Turkey. Ankara Üniversitesi Veteriner Fakültesi Dergisi.

[24] Najafi H, Langeroudi AG, Hashemzadeh M, Karimi V, Madadgar O, Ghafouri SA, Maghsoudlo H, Farahani RK (2016). Molecular characterization of infectious bronchitis viruses isolated from broiler chicken farms in Iran, 2014–2015. Arch Virol.

[25] Seger W, GhalyanchiLangeroudi A, Karimi V, Madadgar O, Marandi MV, Hashemzadeh M (2016). Genotyping of infectious bronchitis viruses from broiler farms in Iraq during 2014–2015. Arch Virol.

[26] Abdel-Moneim AS, Afifi MA, El-Kady MF (2012). Emergence of a novel genotype of avian infectious bronchitis virus in Egypt. Arch Virol.

[27] Susan S, Salama E, Ahmed A (2012). Efficacy of some living classical and variant infectious bronchitis vaccines against local variant isolated from Egypt. Nat Sci.

[28] Abdel-Moneim A, Madbouly H, Gelb J, Ladman B (2002). Isolation and identification of Egypt/Beni-Seuf/01 a novel genotype of infectious bronchitis virus. Vet Med J Giza.

[29] Mahmood ZH, Sleman RR, Uthman AU (2011). Isolation and molecular characterization of Sul/01/09 avian infectious bronchitis virus, indicates the emergence of a new genotype in the Middle East. Vet Microbiol.

[30] Awad F, Forrester A, Baylis M, Lemiere S, Ganapathy K. Protection conferred by live infectious bronchitis vaccine viruses against variant Middle East IS/885/00-like and IS/1494/06-like isolates in commercial broiler chicks. Vet Record Open 2015, 2(2). 10.1136/vetreco-2014-000111 PMC4567785 26392909

[31] Erfanmanesh A, Ghalyanchilangeroudi A, Nikaein D, Hosseini H, Mohajerfar T: Evaluation of inactivated vaccine of the variant 2 (IS-1494 /GI-23) genotype of avian infectious bronchitis. Comp Immunol Microbiol Infect Dis 2020, 71:101497. 10.1016/j.cimid.2020.101497 PMC7260563 32505764

[32] Sultan HA, Ali A, El Feil WK, Bazid AHI, Zain El-Abideen MA, Kilany WH (2019). Protective Efficacy of Different Live Attenuated Infectious Bronchitis Virus Vaccination Regimes Against Challenge With IBV Variant-2 Circulating in the Middle East. Front Vet Sci.

[33] Jackwood MW, de Wit JJ: Infectious Bronchitis. In: Diseases of Poultry 13th edn. Edited by Swayne D, glisson J, McDougald L, Nolan L, Suarez D, Nair V. Ames: Blackwell Publishing Professional; 2013: 139–159.

